# Prevalence of interpersonal trauma exposure and trauma-related disorders in severe mental illness

**DOI:** 10.3402/ejpt.v4i0.19985

**Published:** 2013-04-08

**Authors:** Maria W. Mauritz, Peter J. J. Goossens, Nel Draijer, Theo van Achterberg

**Affiliations:** 1 Community Mental Health Care Unit/Long Treatment, GGNet, Warnsveld, The Netherlands; 2 Scientific Institute for Quality of Healthcare, Radboud University Nijmegen Medical Centre, Nijmegen, The Netherlands; 3 Saxion University of Applied Sciences, Expertise Centre of Health, Social Work & Technology, Deventer, The Netherlands; 4 Specialist Centre for Bipolar Disorders, Dimence, Deventer, The Netherlands; 5 Department of Psychiatry, VU University Medical Center, Amsterdam, The Netherlands

**Keywords:** Childhood trauma, sexual abuse, physical abuse, emotional abuse, neglect, posttraumatic stress disorder, complex posttraumatic stress disorder, dissociative disorder, severe mental illness

## Abstract

**Background:**

Interpersonal trauma exposure and trauma-related disorders in people with severe mental illness (SMI) are often not recognized in clinical practice.

**Objective:**

To substantiate the prevalence of interpersonal trauma exposure and trauma-related disorders in people with SMI.

**Methods:**

We conducted a systematic review of four databases (1980–2010) and then described and analysed 33 studies in terms of primary diagnosis and instruments used to measure trauma exposure and trauma-related disorders.

**Results:**

Population-weighted mean prevalence rates in SMI were physical abuse 47% (range 25–72%), sexual abuse 37% (range 24–49%), and posttraumatic stress disorder (PTSD) 30% (range 20–47%). Compared to men, women showed a higher prevalence of sexual abuse in schizophrenia spectrum disorder, bipolar disorder, and mixed diagnosis groups labelled as having SMI.

**Conclusions:**

Prevalence rates of interpersonal trauma and trauma-related disorders were significantly higher in SMI than in the general population. Emotional abuse and neglect, physical neglect, complex PTSD, and dissociative disorders have been scarcely examined in SMI.

Trauma exposure and posttraumatic stress disorder (PTSD) have been receiving growing attention in research over the last decades. Despite this, trauma exposure and PTSD are significantly overlooked in the treatment of patients with severe mental illness (SMI): documentation of trauma and symptoms of trauma is exceptionally low in the medical records of patients with SMI. Improved recognition is thus needed to provide adequate treatment and meaningful services for this vulnerable population (Cusack, Grubaugh, Knapp, & Frueh, 2006; Mueser et al., 2004; van den Berg & van der Gaag, 2012).

One reason for the poor recognition of trauma in patients with SMI is the overlap which often occurs between the symptoms of trauma-related disorders and the symptoms of SMI. For instance, dissociation and psychotic symptoms can be signs of both PTSD and schizophrenia (Kilcommons & Morrison, 2005; Lysaker & LaRocco, 2008; van Gerven, Hart, Nijenhuis, & Kuipers, 2002). Another reason for poor recognition of trauma in patients with SMI is that many clinicians have been hesitant to pay attention to traumatic experiences in the past because they think that this could lead to further distress and impairment. There is, however, no evidence for this conviction (Cusack et al., 2006; Griffin, Resick, Waldrop, & Mechanic, 2003). Finally, the majority of patients with SMI entered the mental health care system a long time ago when knowledge of the role of traumatic experiences in the onset and course of these disorders was limited. Among the traumatic experiences, repeated interpersonal trauma, such as emotional, physical, and sexual abuse, can lead to both PTSD as well as more severe trauma-related disorders like complex PTSD and dissociative disorders. It has further been documented that a history of interpersonal trauma exposure and the existence of trauma-related disorders negatively affects the course of SMI (Friedl & Draijer, 2000; Mueser, Rosenberg, Goodman, & Trumbetta, 2002; Mueser et al., 2004). Despite this, the prevalence of interpersonal trauma exposure and trauma-related disorders in people with SMI has been scarcely investigated.

In order to gain insight into interpersonal trauma exposure and trauma-related disorders among patients with SMI, we conducted a systematic review of the relevant research literature with the following research question in mind.

What is the prevalence of interpersonal trauma exposure and trauma-related disorders in patients with severe mental illness (SMI) receiving mental health care?

As SMI and trauma-related disorders are the central concepts in this review, their definitions—which are still developing—will first be considered below.

## Severe mental illness

The concept of SMI often lacks clarity and specificity. “SMI” is the abbreviation commonly used for the following: severe mental illness, serious mental illness, or severe and persistent mental illness. When Schinnar, Rothbard, and Kanter (1990) compared 17 definitions of SMI used in the USA between 1972 and 1987, they decided that the optimal definition was the one provided by the National Institute of Mental Health (NIMH) with the following criteria: a diagnosis of non-organic psychosis or personality disorder; duration in terms of prolonged illness and long-term treatment; and disability including three of eight criteria: (a) social behaviour demanding a mental health intervention; (b) limited ability to obtain assistance; (c) impaired activities of daily living and basic needs; (d) impaired social functioning; (e) impaired performance in employment or (f) non-work (homemaking); (g) vulnerability to stress; and (h) disability that causes dependency (National Institute of Mental Health [NIMH], 1987; Schinnar et al., 1990).

Kessler et al. (2003) defined SMI as any 12-month DSM-IV disorder, other than a substance use disorder, with a global assessment of functioning (GAF) score<60. In Europe, Ruggeri, Leese, Thornicroft, Bisoffi, and Tansella (2000) have operationalized the NIMH criteria for SMI along two dimensions: duration of treatment (≥2 years) and degree of dysfunction (GAF-score ≤50 or 70 depending on the primary diagnosis) for any mental disorder.

## Posttraumatic stress disorder

In the DSM-IV-TR, posttraumatic stress disorder (PTSD) is classified as an anxiety disorder which follows a traumatic event and entails intense fear and/or feelings of helplessness. Symptoms are divided into three clusters: (a) intrusions in the form of re-experiencing the trauma via nightmares, obsessive thoughts and/or flashbacks; (b) avoidance of situations, people and/or objects which remind the patient of the traumatic event; and (c) increased anxiety in general, possibly with a heightened startle response and alertness (APA, 2000).

## Complex PTSD

PTSD captures only a limited aspect of posttraumatic psychopathology, especially in victims with severe and prolonged trauma. Herman (1992) has identified seven symptom clusters for these victim groups: dysregulation of (a) affect and impulses; (b) attention or consciousness; (c) self-perceptions; (d) perceptions of the perpetrator; (e) relations with others; (f) somatization; and (g) systems of meaning. This constellation of symptom clusters is referred to as complex PTSD or disorder of extreme stress not otherwise specified (DESNOS) but classified in the DSM-IV as PTSD with associated features (APA, 2000; Herman, 1992; Pelcovitz et al., 1997; van der Kolk, Roth, Pelcovitz, Sunday, & Spinazzola, 2005).

Bryant (2010) has argued that emotion dysregulation is the core feature of complex PTSD. Dysregulated affect and impulses, self-perceptions and relations with others are also highly characteristic of personality disorders, particularly borderline personality disorder (BPD) (APA, 2000; Herman, Perry, & van der Kolk, 1989). This makes BPD difficult to distinguish from complex PTSD. Lewis and Grenyer (2009) reviewed the on-going controversy on the nature of the relationship between complex PTSD and BPD concluded that these disorders often cohere, but are separate phenomena.

## Dissociation and dissociative disorders

It has been further argued in the literature that dissociation is a core feature of complex PTSD. Van der Hart, Nijenhuis, and Steele (2005) have postulated that traumatization essentially involves some degree of division or dissociation of the psychobiological systems which constitute identity. Dissociative identity disorder (DID) and dissociative disorder not otherwise specified (DDNOS) have indeed been found to be highly correlated with a history of severe and prolonged traumatic experiences (82%) and therefore were considered as trauma-related disorders in such cases (Boon & Draijer, 1993; Draijer & Boon, 1990; Sar, 2011).

Based on this overview of concepts, we used the following definitions in our review: SMI was defined as any mental disorder, other than a substance use disorder, with the following two dimensions: duration of mental illness and obvious dysfunction. PTSD, complex PTSD, DID, and DDNOS were considered as trauma-related disorders. BPD was considered as a separate disorder.

## Methods

### Operationalization of concepts

In this review, the two dimensions of *SMI* were operationalized as (1) duration of mental illness ≥ 2 years (Ruggeri et al., 2000) and (2) obvious dysfunction as a GAF-score ≤60 or a clear description of a minimum of three disabilities according to the NIMH criteria for SMI (Kessler et al., 2003; NIMH, 1987). *Interpersonal trauma* was defined as involving any of the following traumatic experiences: emotional abuse (EA), emotional neglect (EN), physical abuse (PA), physical neglect (PN), and/or sexual abuse (SA) in childhood and/or adulthood. *Trauma-related disorders* were considered present when a diagnosis had been made of PTSD, PTSD with associated features, complex PTSD, DESNOS, DID, and/or DDNOS.

### Search

The Medline, PsycINFO, Embase, and CINAHL databases were searched for the period between 1980 and 2010. The subject headings and keywords were as follows. For *SMI*, schizophrenia/psychotic disorders, bipolar disorders, major (chronic) depression/major depressive disorders, anxiety disorders, eating disorders, and personality disorders. For *interpersonal trauma exposure*: child abuse, emotional abuse; emotional neglect; physical abuse; physical neglect; sexual abuse; physical assault; and sexual assault. For *trauma-related disorders*: PTSD, complex PTSD, DESNOS, and dissociative disorders. *Prevalence* was searched using the following terms: prevalence, co-morbidity, and risk factors.

All of the keywords for trauma and prevalence were first combined. These words were then combined with each mental disorder. The exact search strategy is outlined in Supplement 1.

### Selection criteria

*Inclusion criteria* for peer-reviewed publications using adult study populations (≥18 years) were as follows:

1. SMI was labelled as such or one of the following mental disorders was mentioned: schizophrenia spectrum disorders, bipolar disorders, major depression/depressive disorders, anxiety disorders, eating disorders, or personality disorders
  - Classified in DSM-III, DSM-IV or DSM-IV-tr
  - *SMI* determined on the basis of both dimensions:
    - Duration of illness and/or treatment (≥2 years)
    - Obvious dysfunction (GAF ≤60 or clear description of minimum of three impairments according the NIMH definition)
  *and*
2. Report of prevalence of interpersonal trauma exposure with specific description:
  - Type of abuse, reference period (childhood, adulthood, lifetime)
  - Description of the diagnostic instruments used
  *and*
3. Report of prevalence of *trauma-related disorders*:
  - PTSD, complex PTSD (PTSD with associated features or DESNOS), DID, and/or DDNOS
  - Relevant diagnostic instruments were described

The *exclusion criteria* for our review were as follows:

1. Substance use disorders as only diagnosis
2. Developmental disorders; delirium, dementia, amnesia or other cognitive disorders with a physical origin
3. Forensic and/or imprisoned populations

### Selection

**Fig. 1:**
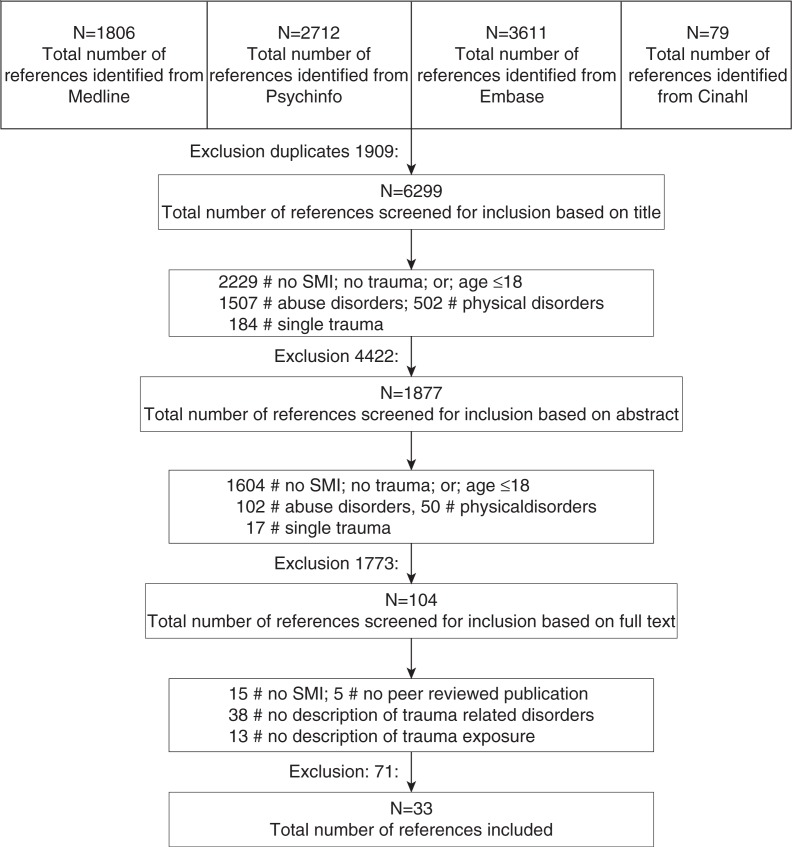
Selection in scheme.

Figure 1 shows the selection of the publications for this review. Of the initial 6,299 unique hits, 4,422 publications were excluded because the title did not mention SMI and/or interpersonal trauma or trauma-related disorders, or the title mentioned the age ≤18. The abstracts from the remaining 1,877 publications were independently reviewed by two researchers. Of these, 104 described the prevalence of interpersonal trauma exposure or trauma-related disorders in SMI and the entire publication was therefore reviewed by three researchers independently. In the end, 33 publications described the prevalence of *both* trauma exposure and trauma-related disorders in SMI and were thus selected for inclusion in the review.

Those studies in which SMI was explicitly mentioned only included the following disorders: schizophrenia (spectrum disorder), bipolar disorder, major depressive disorder, and personality disorder. Anxiety disorders and eating disorders were never characterized as SMI in the selected publications and contained insufficient information about the duration of illness and obvious dysfunction. For this reason, anxiety or eating disorder as the primary diagnosis were excluded from our review although the prevalence of trauma in such disorders is notable (Brewerton, 2007; Grant, Beck, Marques, Palyo, & Clapp, 2008; Stein et al., 1996). One study of bipolar disorders was also excluded from our review due to the exclusion of the severely ill subgroup of patients (Brown, Mc Bride, Bauer, & Williford, 2005).

### Data extraction

The following data were extracted from the 33 publications included in the review:

1. Population characteristics: gender, age, country, care setting, research type
2. Primary diagnosis for SMI
3. Diagnostic instruments used
4. Prevalence rates for interpersonal trauma: type (EA, EN, PA, PN, SA), reference period (childhood, adulthood, lifetime)
5. Prevalence rates for trauma-related disorder: description of diagnosis
6. General conclusions of the study

### Quality assessment

The following quality aspects of the studies included in the review were assessed:

1. Study design
2. Sampling: inclusion and exclusion criteria; (non) response
3. Use of diagnostic instruments to determine primary diagnosis (SMI)
4. Use of diagnostic and research instruments to measure trauma exposure and trauma-related disorders

## Analysis

Prevalence rates were determined according to diagnostic group and gender. To facilitate comparison of the prevalence rates, population-weighted means were calculated for each of the diagnostic groups. This was also done for gender when reported. A formal meta-analysis was not performed because of the wide variety of objectives and research designs in the studies, the heterogeneity of the instruments used to assess interpersonal trauma exposure and the affected stage of life, e.g., childhood, adulthood, or lifetime.

## Results

In the 33 studies included in our review, the following disorders were addressed: bipolar disorder (8), major depressive disorder (6), schizophrenia spectrum disorder (7), personality disorder (5), and mixed diagnostic groups, explicitly labelled as SMI (7). All publications described the prevalence of interpersonal trauma exposure, and all but one (Ross & Keyes, 2004) described the prevalence of PTSD. One publication described the prevalence of complex PTSD (Barnow, Plock, Spitzer, Hamann, & Freyberger, 2005) and two reported the prevalence of DID and/or DDNOS (Ross & Keyes, 2004; Sar et al., 2003). The characteristics of the studies are outlined in Table 1.

**Table 1:** Study design, population characteristics, and instruments used in the 33 studies of this review

											Instruments for assessing		
											
	Author	Study design	Country	*N*	SC	Response	Mean age/SD or range	Setting	Diagnosis/disorder	TI	Diagnosis	Trauma exposure	PTSD
Bipolar disorder													
1	Assion et al., 2009	Prevalence study	Germany	*N*= 74 (*f*=44)	Y		48.3 (13)	In- and outpatient	Bipolar-I: euthymic/mild/mod. depressed	Y	MINI/HAM-D/YMRS	PDS	PDS/CAPS
2	Goldberg & Garno, 2005	Prevalence study	USA	*N*=100 (*f*=49)	N		42.6 (13.2) 36.6 (9.7) (T)	Inpatient	Bipolar-I (73) Bipolar-II (27)	N	SCID-I/HAM-D/YRMS	CTQ/TSS	SCID-I
3	Kauer et al., 2007	Correlation study	Brazil	*N*= 163 (*f*=117)	N		42.5 (SD 11.6)	Outpatient	Bipolar-I Bipolar-II	N	Chart DSM-IV/HDRS/YMRS	SCID-I (criteria A1 and A2)	SCID-I-PTSD module
4	Leverich et al., 2002ab	Correlation study	USA	*N*=651 (*f*=274)	N		41 (12)	Outpatient	Bipolar-I (475) Bipolar-II (137) Bipolar NOS (12)	N	SCID-P/IDS/YMRS	Patient Quest. with items: EA, PA, SA	SC-I
5	Maguire et al., 2008	Prevalence study	N Ireland (UK)	*N*=60 (*f*=34)	Y	Non-response 24; drop-out 15	49 (25–70)	Outpatient	Bipolar-I Bipolar-II	Y	Chart DSM-IV/BDI	CHQ/THQ	PDS
6	Meade et al., 2009	Treatment study	USA	*N*=90 (*f*=41)	Y	Non-response 57; drop-out 42	40.6 (±11.7)	In- and outpatient	Bipolar-I with SUD	Y	SCID-I/HAM-D/YRMS	ASI: 2 items	SCID-I
7	Neria et al., 2005	Correlation study	USA	*N*=109 (*f*=58)	N	Response 88,7%	*m*=23 (15–54) *f*=29 (16–57)	Inpatient	Bipolar with psychotic features	N	SCID-I-DSM-III and IV	CIDI-PTSD/SCID-PTSD	CIDI-PTSD/SCID-PTSD
8	Neria et al., 2008	Prevalence study	USA	*N*=96 (*f*=64)	Y		50 (18–70)	Outpatient	Bipolar spectrum	N	MDQ/PDQ	LES	PCL-C
Major depressive disorder													
9	Bernet et al., 1999	Correlation study	USA	*N*=47 (*f*=24)	Y		39.0	Outpatient	Major depressive disorder	Y	SCID-I/HRSD	CTQ	SCID-I
10	Carlier et al., 2000	Prevalence study	The Netherlands	*N*=69 (*f*=45)	Y		45.4 (12.9)	Outpatient	Major depressive episode	N	SCID-DSM-III-R	LTE	SI-PTSD
11	Gaudiano et al., 2010	Correlation study	USA	*N*=623 (*f*=397)	N		38.5 (12)	Outpatient	Major depressive disorder (psychotic *n*=32)	Y	SCID-I/SIDP-IV/CGI	CTQ	SCID-I
12	Oquendo et al., 2005	Correlation study	USA	*N*=230 (*f*=180)	N		41.7 (0.6)	In- (113) and outpatient (117)	Major depressive episode	Y	SCID-I/SCID-II/HMDRS/BDI	SCID-I	SCID-I
13	Zimmerman et al., 1999	Correlation study	USA	*N*=235 (*f*=152)	N		40.6 (14.03)	Outpatient	Major depression (psychotic *n*=19)	Y	SCID-I	SCID-PTSD	SCID-PTSD
14	Zlotnick et al., 2001	Correlation study	USA	*N*=235 (*f*=152)	N		40.6 (14.0)	Outpatient	Major depression	Y	SCID-I/SIDP/SADS	SCID-PTSD	SCID-PTSD
Schizophrenia spectrum disorder													
15	Beattie et al., 2009	Correlation study	N Ireland (UK)	*N*=47 (*f*=22)	N		37.5 (11.5)	Outpatient <12 months	Schizophrenia (25) Schizoaffective (7) Other psychosis (10)	N	KGV:pos./neg./affect symptoms	CTQ/THQ	IES-R
16	Calhoun et al., 2007	Prevalence study	USA	*N*=165 (*f*=0)	N		48 (7.8)	Inpatient (veterans)	Schizophrenia Schizoaffective	Y	SCID-I	SAEQ/CTS-R/CES	PCL
17	Kilcommons & Morrison, 2005	Prevalence study	UK	*N*=32 (*f*=7)	N	Non-response 3%	34.5 (10.0)	Outpatient	Schizophrenia spectrum	N	Chart DSM-IV/PANSS	THQ	PSS-R
18	Lommen et al., 2009	Prevalence study	Netherlands	*N*=33 (*f*=10)	Y	Non-response; 40 drop-out 2	42.3 (10.6)	Outpatient	Schizophrenia (23)/Schizoaffective (10)	N		THQ-R	PSS-SR/PCTI
19	Resnick et al., 2003	Correlation study	USA	*N*=47 (*f*=30)	N		44.1 (9.7)	Outpatient	Schizophrenia (39) Schizoaffective (8)	Y	SCID-I/PANSS	THQ-R	CAPS
20	Rosenberg et al., 2007	Correlation study	USA	*N*=569 (*f*=183)	N		42 (9.0)	Outpatient	Schizophrenia (288) Schizoaffective (98)	N	Chart DSM-IV (81%)/SCID (19%)	SAEQ/CTS	PCL
21	Ross & Keyes, 2004	Prevalence study	USA	*N*=60 (*f*=23)	N	Non-response 3	40.1 (13.4)	In- and outpatient	Schizophrenia	N	Chart DSM III-R/SAPS/SANS	Not specified	DDIS/DES-T
Personality disorder													
22	Barnow et al., 2005	Prevalence study	Germany	*N*=51 (*f*=44)	Y		26.5 (±7.6)	Inpatient	Borderline personality	Y	SCID-II/BPI/DIA-X (CIDI)	SCID-I-PTSD	SCID-I-PTSD/SIDES
23	van den Bosch et al., 2003	Correlation study	The Netherlands	*N*=63 (*f*=63, addicted)	Y		34.9 (7.7)	Inpatient	Borderline personality (addicted 34)	Y	SCID-II/PDQ4+	STI	SCID-I-PTSD
24	Sar et al., 2003	Prevalence study	Turkey	*N*=25 (*f*=20)	Y	Response 79,5%	30.0	Outpatient	Borderline personality	Y	SCID-II (DSM-III-R)	History Form for Childhood Abuse and Neglect	SCID-PTSD/DES/DDIS/SCID-D
25	Zlotnick et al., 2003	Correlation study	USA	*N*= 266 (*f*=186)	Y		31.5 (7.5)	In- and outpatient	Borderline personality	N	DIPD-IV/SCID-I/SNAP	CEQ	SCID-I/DIPD-IV
26	Yen et al., 2002	Correlation study	USA	*N*=653 (*f*=324)	Y	Dropout 15	32.8 (8.0)	In- and outpatient	Borderline (176, *f*=123) Schizotypal (86, *f*=39) Avoidant (153, *f*=100) Obs. Compulsive (153, *f*=62)	N	DIPV-IV/SCID-I	SCID-I trauma module	SCID-I trauma module
Severe mental illness													
27	Cusack et al., 2006.	Prevalence study	USA	*N*=142 (*f*=63)	Y		46.2 (11.6)	Outpatient	Schizophrenia Bipolar Major depression	N	Chart Review Instrument	TAA	PCL
28	Davies-Netzley, Hurlburt, & Hough, 1996	Correlation study	USA	*N*=120 (*f*=120)	Y		37.0 (18–63)	Outpatient	Schizophrenia (57) Bipolar (26) Major Depression (37)	Y	DIS (DSM-III-R)/CES-D	Specified questions about child abuse	PTSD section of DIS
29	Ford & Fournier, 2007	Prevalence study	USA	*N*=35 (*f*=35)	N		41.0 (29–68)	Outpatient	Schizophrenia Bipolar Major depression	Y	SCID-I	TESI	CAPS/SIDES
30	Goodman et al., 1999	Reliability study	USA	*N*=50 (*f*=29)	Y	Response 80%; drop-out 3	*m*=37.6 (7.3) *f*=42.1 (7.6)	Outpatient	Schizophrenia (32) Bipolar (16) Psychotic (2)	N	SCID-I	SAEQ/CTS-2 (PA/SA/injury)	PCL-S
31	Lu et al., 2008	Correlation study	USA	*N*=254 (*f*=112)	N	Non-response; 20	42.9 (11.0)	In- (109) and outpatient (145)	Major depression (100) Bipolar (154)	N	Chart (81.0%) SCID-I (19.0%)	SAEQ/CTS-R	PCL
32	Mueser et al., 1998	Prevalence study	USA	*N*=275 (*f*=153)	N	Non-response 10%	40.0 (11.6)	In- (92) and outpatient (183)	Schizophrenia (64) Schizoaffective (30) Bipolar (50) Depression (65) Borderline pers. (22) Other pers. (10) Other diagnosis (34)	Y	Chart (81.0%) SCID-I (19.0%)	THQ (childhood and adulthood separated asked)/CVS	SCID/PCL-S
33	Mueser et al., 2004	Prevalence and correlation study	USA	*N*=782 (*f*=321)	N	Response 87%	age≥18	Outpatient	Schizophrenia (363) Schizoaffective (163) Bipolar (141) Major depression (78) Other diagnosis (26)	N	Chart (81.0%) SCID-I (19.0%)	SAEQ/CTS (child)/CTS-2 (adult)	PCL

SC: Selection criteria other than inclusion criteria.

TI: Trained interviewers.

### Quality of the included studies

All 33 publications fulfilled the required inclusion criteria for primary diagnosis and age. Eight publications described additional selection criteria and also mentioned training of the diagnostic interviewers (Table 1). The other publications described only additional selection criteria or the training of the interviewers but not both. The instruments used for the primary diagnosis in the 33 studies are reported in Table 1 and described further in Supplement 2. The diagnostic instruments used to assess trauma exposure and trauma-related disorders are similarly reported in Table 1 and described further in Supplement 3.

### Prevalence of PA, SA, and PTSD according to diagnostic group and reference period

**Fig. 2:**
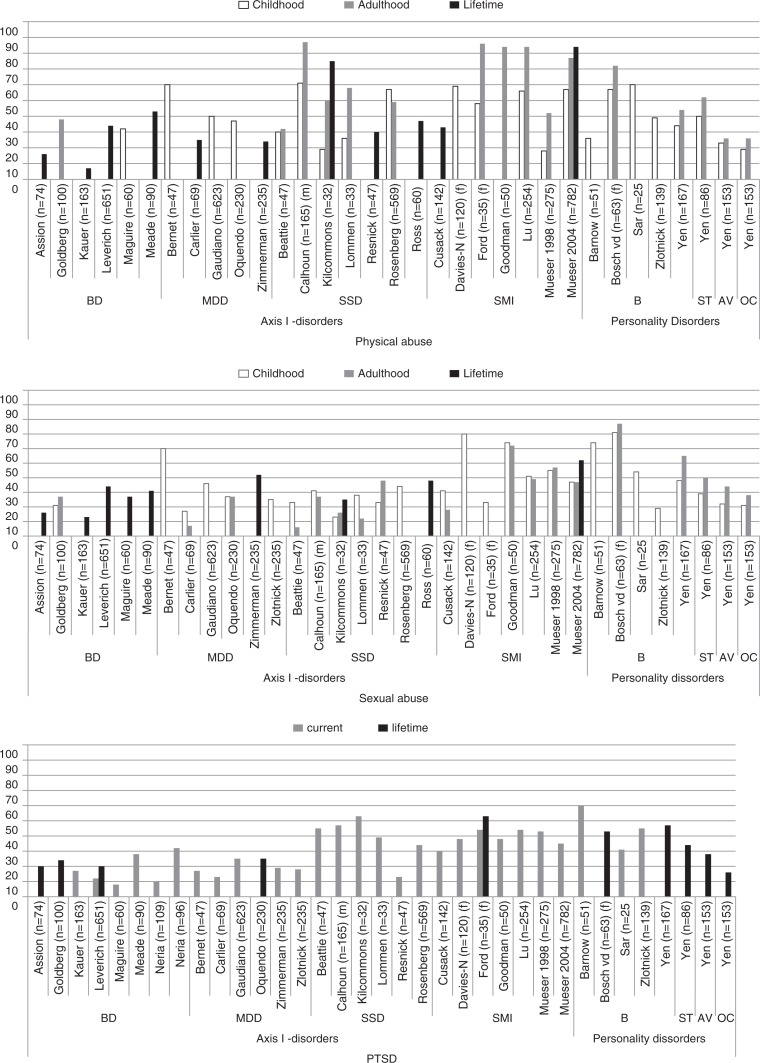
Prevalence rates for physical abuse, sexual abuse, and PTSD by disorder and reference period. BD, bipolar disorder; MDD, major depressive disorder; SSD, schizophrenia spectrum disorder; SMI, severe mental illness; B, borderline; ST, schizotypal; AV, avoidant; OC, obsessive compulsive (personality disorder).

Prevalence rates of PA and SA were examined in all 33 studies according to reference period (i.e., childhood only, adulthood only, both childhood and adulthood, or lifetime) (Fig. 2). Two studies considered PA and SA together and could not be shown in the figures. These combined lifetime prevalence rates were 40 and 53%, respectively (Neria, Bromet, Carlson, & Naz, 2005; Neria et al., 2008).

In those studies reporting on both the adult and childhood prevalence of PA, adult PA tended to be higher than childhood PA for schizophrenia spectrum disorders, personality disorders and SMI (mixed diagnoses group). Similarly, the adult prevalence of SA tended to be higher than the childhood prevalence for SMI and personality disorders (Fig. 2).

### Differences in prevalence of PA, SA and PTSD across diagnostic groups

**Fig. 3:**
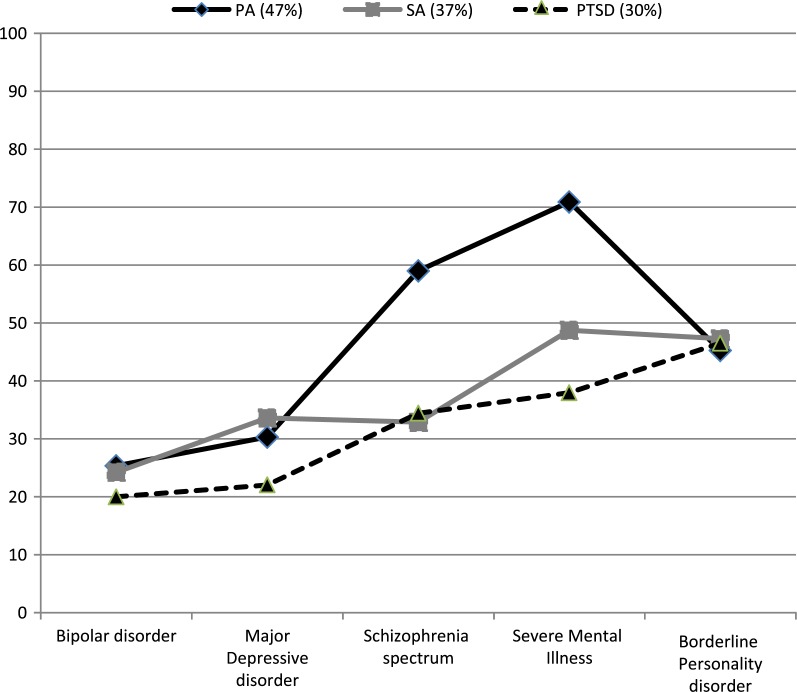
Overview of mean prevalence of physical abuse, sexual abuse, and PTSD by diagnostic group.

Figure 3 shows the mean prevalence rates for PA, SA, and PTSD weighted according to population size. When more than one rate was given for a particular type of trauma exposure (e.g., both childhood and adulthood), the highest prevalence rate was adopted. Prevalence rates for PA and PTSD were relatively low in bipolar and major depressive disorder. The mean prevalence of PA was most high in schizophrenia and SMI while the mean prevalence of SA and PTSD was most high in BPD and SMI.

### Prevalence of PA, SA and PTSD according to diagnostic group and gender

**Fig. 4:**
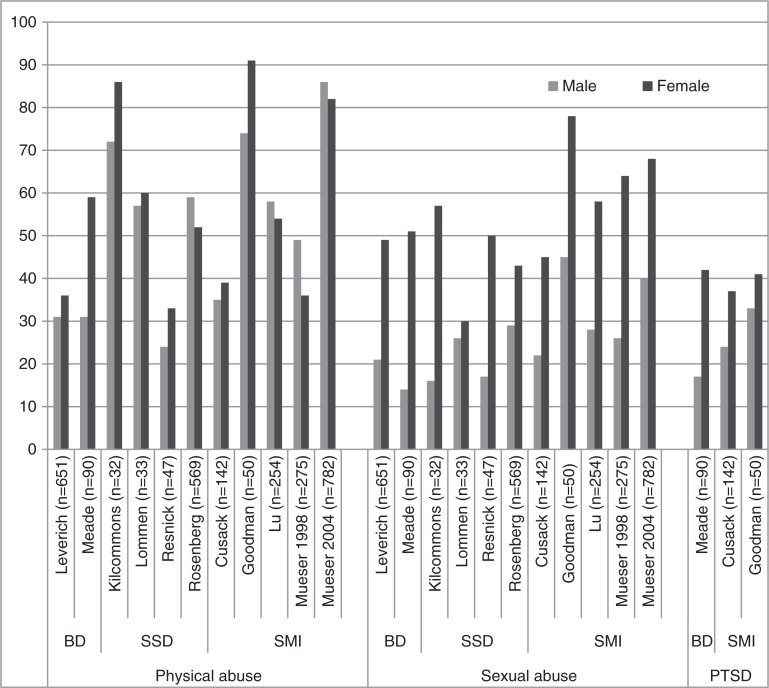
Prevalence rates for physical abuse, sexual abuse, and PTSD by disorder and gender. BD, bipolar disorder; SSD, schizophrenia spectrum disorder; SMI, severe mental illness.

The prevalence of PA and SA according to gender was examined in 11 studies and involved the following diagnostic groups: bipolar disorder, schizophrenia spectrum disorder, and SMI. Three of these studies also described prevalence according to gender for PTSD (Fig. 4). Prevalence rates for SA were significantly higher for women than for men, but less so for PA.

### Prevalence of EA, EN, and PN according to diagnostic group and reference period

Estimates of the prevalence of EA in bipolar disorder were provided in only two studies: 43% for childhood (Maguire, Mc Cusker, Meenagh, Mulholland, & Shannon, 2008) and 6% for lifetime (Kauer-Sant’ Anna et al., 2007). This large difference could reflect differences in the diagnostic instruments used in these studies (see Table 1). The childhood prevalence of EA in major depressive disorder was reported to be 39% by Gaudiano and Zimmerman (2010) and 49% by Bernet and Stein (1999). The prevalence of childhood EA was highest in the psychotic subgroup of major depression with 53% (Gaudiano & Zimmerman, 2010). In a single study of schizophrenia, the childhood prevalences of EA, EN, and PN were found to be 13, 13, and 6%, respectively (Beattie, Shannon, Kavanagh, & Mulholland, 2009). In BPD, the prevalence of childhood EA was 60–63% (Sar et al., 2003; Zlotnick et al., 2003). The prevalence of total neglect in BPD was 60–88% (Sar et al., 2003; van den Bosch, Verheul, Langeland, & van den Brink, 2003; Zlotnick et al., 2003).

### Complex PTSD and dissociative disorders

Complex PTSD in schizophrenia was investigated and present in one study, but the prevalence rate was not reported (Ford & Fournier, 2007). The current prevalence of complex PTSD in BPD was 31% (Barnow et al., 2005).

The prevalence of DID in schizophrenia has been reported to be 16% (Ross & Keyes, 2004). And the current prevalence of DID in BPD has been reported to be 24% and that of DDNOS in BPD to be 36% (Sar et al., 2003). In this study, DID only appeared in patients with both BPD and a history of trauma exposure and it also correlated with the highest prevalence of childhood trauma.

### Notable clinical findings

#### Co-morbidity

Information on co-morbid conditions was reported in 20 publications. Of these, 13 compared co-morbidity for traumatized versus not traumatized subgroups and found a higher degree of co-morbidity for Axis I and/or Axis II disorders for all of the traumatized subgroups. The prevalence of co-morbid substance use disorders was also significantly higher for the traumatized subgroups and negatively influenced the course of mental illness (Barnow et al., 2005; Bernet & Stein, 1999; Calhoun et al., 2007; Carlier, Voerman, & Gersons, 2000; Ford & Fournier, 2007; Goldberg & Garno, 2005; Leverich et al., 2002a; Leverich, Perez, Luckenbaugh, & Post, 2002b; Lu et al., 2008; Meade et al., 2009; Oquendo et al., 2005; Rosenberg, Lu, Mueser, Jankowski, & Cournos, 2007; Ross & Keyes, 2004; Zlotnick, Mattia, & Zimmerman, 2000).

### Influence of interpersonal trauma exposure and trauma-related disorders on the course of mental illness and severity of symptoms

Childhood SA and childhood EA were associated with an 8–9 year earlier onset of illness in major depressive disorder (Bernet & Stein, 1999; Zlotnick et al., 2000). Childhood PA and childhood SA were strongly associated with PTSD in psychotic subtypes of major depressive disorder (Gaudiano & Zimmerman, 2010; Zimmerman & Mattia, 1999). Childhood trauma exposure had a negative effect on the course of illness in schizophrenia spectrum disorder (Rosenberg et al., 2007). Severity of trauma exposure was associated with positive symptoms in schizophrenia (Resnick, Bond, & Mueser, 2003; Ross & Keyes, 2004; Kilcommons & Morrison, 2005). SMI patients with a history of both PA and SA attempted suicide five times more frequently (Ford & Fournier, 2007). The number of types of trauma exposure also predicted PTSD in SMI (Mueser et al., 1998).

Co-morbid lifetime PTSD predicted a worse clinical outcome for bipolar disorder: a 6-year earlier start of the symptoms, more severe symptoms, more suicide attempts and ultra-rapid cycling of mood swings (Assion et al., 2009; Kauer-Sant’ Anna, 2007; Leverich et al., 2002a, b; Meade et al., 2009). PTSD was associated with more severe symptoms and more suicide attempts for major depressive disorder (Oquendo et al., 2005); it was also four times more present for the psychotic subtypes of major depressive disorder than for the non-psychotic subtypes (Gaudiano & Zimmerman, 2010; Zimmerman & Mattia, 1999). PTSD related significantly to Axis I co-morbidity and severe emotion dysregulation in BPD (Zlotnick et al., 2003). Complex PTSD, DID, and DDNOS related significantly to a chronic course of illness, severe clinical conditions, more self-destructive behaviour and suicide attempts in BPD (Barnow et al., 2005; Sar et al., 2003).

## Discussion

### Main findings

The mean prevalence rates in SMI ranged according to the diagnostic group were as follows: PA 47% (range 25–72%), SA 37% (range 24–49%), and PTSD 30% (range 20–47%). Compared to men, women showed a higher prevalence of SA in schizophrenia spectrum disorder, bipolar disorder, and mixed diagnosis groups labelled as having SMI.

The prevalence rates in SMI were significantly higher than those in the general population. More specifically, the prevalence in SMI as compared to the general population was 47% versus 21% for PA and 37% versus 23% for SA. Gender differences in the prevalence of SA in SMI (20–34% for men, 44–64% for women) were roughly comparable to that in the general population (14% for men, 32% for women) (Briere & Elliott, 2003). The PTSD prevalence of 30% in SMI was also significantly higher than the PTSD prevalence of 7% among a general population of adult Americans (Kessler, Berglund, Demler, Jin, & Walters, 2005a; Kessler, Chiu, Demler, Merikangas, & Walters, 2005b).

A 35% prevalence of complex PTSD in BPD was reported in one study and distinguished from a 61% prevalence of PTSD (Barnow et al., 2005). The prevalence of complex PTSD (DESNOS) in the general population has been scarcely examined, so further comparison was not possible. One study nevertheless reported a prevalence of 1.0% (Ford, Stockton, Kaltman, & Green, 2006). The prevalence of DID was reported to be 16% in schizophrenia (Ross & Keyes, 2004) and 24% in BPD; for DDNOS, this rate was reported to be 36% in BPD (Sar et al., 2003). The prevalence of DID in the general population has been found to vary from 0.4 to 3.1%, depending on the assessment instrument used (Friedl & Draijer, 2000; Sar, 2011).

### Validity

Our literature search covered a period of 30 years and was conducted independently on four databases. The publications included in the review were selected by three independent reviewers. The primary diagnoses for the patients in the studies we reviewed were usually confirmed via structured interviews like the SCID-I, SCID-II, or MINI (see Supplement 2). In three of the 33 studies, only 19% of the chart diagnoses were confirmed using the SCID-I; in six of the studies, only the chart diagnosis were used. The severity of symptoms was screened for in 12 of the studies—mostly for bipolar disorder (HAM-D, YMRS), major depressive disorder (HAM-D, BDI), and sometimes schizophrenia spectrum disorder (PANSS) (see Table 1).

Trauma history was assessed in the 33 studies we reviewed using 19 different instruments. Sufficient information was generally provided by the different instruments although they differed with regard to the questions used to assess life stage and type of interpersonal trauma. As a result, the prevalence rates were difficult to compare across studies, and the population-weighted prevalence rates should be interpreted with caution.

The quality of the information provided about sampling and severity of illness differed across the studies. In the mixed diagnoses groups characterized as SMI schizophrenia spectrum disorders and/or other psychotic disorders were highly represented (approximately 60%). Almost certainly, these mixed diagnoses groups consisted of the most impaired patients showing high co-morbidity, a worse course of illness, and the highest prevalence of trauma exposure and PTSD.

### Possible limitations

The aim of our literature search could not easily be related to a limited set of search terms. The search strategy we used was exhaustive but may nevertheless not have captured all relevant studies. Nearly all studies were carried out in the USA or Western Europe, with the exception of one in Brazil (Kauer-Sant’ Anna, 2007). Inclusion of only Dutch, English, French, and German language publications may have led to a loss of information.

The aim of our review was to identify the prevalence of interpersonal trauma exposure and trauma-related disorders in SMI. Thus, exclusion of publications which attended to *only* interpersonal trauma exposure or *only* trauma-related disorders may also have led to a loss of relevant information. Some publications only reported regression coefficients but not the original prevalence rates and could not be included.

### Trauma, psychosis, and dissociation

PA and SA in childhood and adulthood were markedly high in psychotic disorders (PA 59%, range 32–87%; SA 33%, range 23–38%). In the SMI group, PA was 71% (range 33–86%) and SA was 49% (range 23–70%). In the psychotic subgroups of major depressive disorder, childhood PA was high (53–60%) and childhood SA was even higher (59–60%) (Gaudiano & Zimmerman, 2010; Zimmerman & Mattia, 1999). Janssen et al. (2004) also found that early childhood trauma such as PA and SA increased the risk of developing positive psychotic symptoms in adulthood in a dose–response manner. The prevalence of PTSD was 34–48% in psychotic disorders and 58–65% in psychotic subgroups of major depressive disorder.

The relationship between trauma and psychosis is nevertheless not straightforward. Trauma can certainly cause psychosis, but psychosis can conversely cause PTSD and both psychosis and PTSD can be part of a spectrum of responses to a traumatic event (Morrison, Frame, & Larkin, 2003; Seedat, Stein, Oosthuizen, Emsley, & Stein, 2003).

Two of the 33 studies included in our review attended to psychosis as an internal threat and causal factor in the occurrence of PTSD. “Internal threat” is not considered an A1-criterion for PTSD in the DSM-IV, but Lommen and Restifo (2009) have reported a prevalence rate of 39% including internal threat and 18% excluding internal threat for PTSD. Resnick et al. (2003) have similarly reported a prevalence rate of only 13% excluding internal threat. The remaining four studies attending to psychosis and schizophrenia did not report on the A1-criterion for PTSD and the extent to which internal threat related to PTSD in these studies was therefore unclear.

Psychosis and dissociation are hard to distinguish in clinical practice (Kilcommons & Morrison, 2005; Lysaker & LaRocco, 2008; van Gerven et al., 2002). When Ross and Keyes (2004) examined dissociation in schizophrenia, those patients showing dissociation were found to have more severe trauma histories, more co-morbidity, and higher scores for both positive and negative symptoms of schizophrenia when compared to patients without dissociation. Read, van Os, Morrison, and Ross (2005) have further stated in their review of childhood trauma, psychosis, and schizophrenia that hallucinations are strongly related to abuse in childhood and more or less memories of the traumatic events. Conceptualization of hallucinations as dissociative memories of traumatic events may thus be valuable for understanding the relations between trauma, psychosis and dissociation. And viewed from this perspective, “Schneiderian and other positive psychotic symptoms follow logically from the existence of a structurally dissociated psyche”. McCarthy-Jones and Davidson (2012) have also recently described the lack of love experienced by abused people. Traumatic experiences may lead to auditory hallucinations with critical voices related to frightening experiences and supportive voices related to lack of love and support.

Psychotic symptoms may thus cohere or fall in with dissociative symptoms and possibly be the result of the same underlying mechanism. Within this context, however, Moskowitz (2011) points to conflicting paradigms: the trauma-dissociation paradigm which is focussed on life events versus the neo-Kraepelinian paradigm which is genetically and biologically based. Moskowitz (2011) suggests that the integration of the paradigms into a broader bio-psycho-social paradigm could be more useful for both future research and treatment.

### Implications of our findings

In clinical practice, assessing interpersonal trauma exposure and trauma-related disorders in SMI can reduce current underreporting and lack of treatment. Trauma history can be assessed without further impairment of the patient (Cusack et al., 2006; Griffin et al., 2003; Tucker, 2002). Patient reports of trauma have also been shown to be reliable (Goodman et al., 1999; Mueser et al., 2004).

PTSD in SMI can be treated effectively using cognitive behavioural therapy: cognitive restructuring (Marcello, Hilton-Lerro, & Mueser, 2009; Mueser et al., 2008; Lu et al., 2009), EMDR (van den Berg & van der Gaag, 2012), and prolonged exposure (van Minnen, Harned, Zoellner, & Mills, 2012). For the treatment of complex PTSD, starting with stabilizing interventions prior to EMDR or prolonged exposure has been shown to improve the overall treatment effect (Cloitre et al., 2010; Dorrepaal et al., 2010, 2012). Assessment and treatment of dissociative disorders in traumatized groups is called for in light of the severe clinical condition of such patients and their low treatment success when dissociative disorders are not recognized (Boon & Draijer, 1993; Friedl & Draijer, 2000; Hart et al., 2005; Moskowitz, 2011; Read et al., 2005; Ross & Keyes, 2004; Sar et al., 2003).

In future research, greater attention should be paid to PN, EN, and EA because these factors have been found to increase the risk of developing PTSD (Beattie et al., 2009; Bernet & Stein, 1999; Gaudiano & Zimmerman, 2010; Maguire et al., 2008; Sar et al., 2003). Complex PTSD and DID should be given greater attention in light of their negative influence on the course of a patient's mental illness and quality of life (Barnow et al., 2005; Read et al. 2005; Ross & Keyes, 2004; Sar et al., 2003).

## Conclusion

The prevalence of interpersonal trauma exposure and PTSD in severely mentally ill patients is significantly higher than in the general population. For PA, SA, and PTSD, the prevalence rates differ depending on the type of mental disorder: lower rates of trauma are found for bipolar and major depressive disorder but higher rates are found for schizophrenia, BPD, and groups labelled as severely mentally ill. EA, EN, PN, complex PTSD, and dissociative disorders have been scarcely examined in SMI. Traumatized patients with SMI show more severe symptoms and a worse course of illness than non-traumatized patients with SMI.

## References

[CIT0001] American Psychiatric Association (2000). Diagnostic and statistical manual of mental disorders.

[CIT0002] Assion H.-J, Brune N, Schmidt N, Aubel T, Edel M.-A, Basilowski M (2009). Trauma exposure and post-traumatic stress disorder in bipolar disorder. Social Psychiatry and Psychiatric Epidemiology.

[CIT0003] Barnow S, Plock K, Spitzer C, Hamann N, Freyberger H.-J (2005). Trauma, spirit and qualities of character in patients with borderline personality disorder and complex posttraumatic disorder. Verhaltenstherapie.

[CIT0004] Beattie N, Shannon C, Kavanagh M, Mulholland C (2009). Predictors of PTSD symptoms in response to psychosis and psychiatric admission. The Journal of Nervous and Mental Disease.

[CIT0005] Bernet C. Z, Stein M. B (1999). Relationship of childhood maltreatment to the onset and course of major depression in adulthood. Depression and Anxiety.

[CIT0006] Boon S, Draijer N (1993). Multiple personality disorder in The Netherlands: A clinical investigation of 71 patients. American Journal of Psychiatry.

[CIT0007] Brewerton T. D (2007). Eating disorders, trauma, and comorbidity: Focus on PTSD. Brunner-Mazel Eating Disorders Monograph Series.

[CIT0008] Briere J, Elliott D. M (2003). Prevalence and psychological sequelae of self-reported childhood physical and sexual abuse in a general population sample of men and women. Child Abuse & Neglect.

[CIT0009] Brown G. R, Mc Bride L, Bauer M. S, Williford W. O (2005). Impact of childhood abuse on the course of bipolar disorder: A replication study in U.S. veterans. Journal of Affective Disorders.

[CIT0010] Bryant A (2010). The complexity of complex PTSD. American Journal of Psychiatry.

[CIT0011] Calhoun P. S, Stechuchak K. M, Strauss J, Bosworth H. B, Marx C. E, Butterfield M. I (2007). Interpersonal trauma, war zone exposure, and posttraumatic stress disorder among veterans with schizophrenia. Schizophrenia Research.

[CIT0012] Carlier I. V. E, Voerman B. E, Gersons B. P. R (2000). Intrusive traumatic recollections and comorbid posttraumatic stress disorder in depressed patients. Psychosomatic Medicine.

[CIT0013] Cloitre M, Stovall-McClough K. C, Nooner K, Zorbas P, Cherry S, Jackson C. L (2010). Treatment for PTSD related to childhood abuse: A randomized controlled trial. American Journal of Psychiatry.

[CIT0014] Cusack K. J, Grubaugh A. L, Knapp R. G, Frueh B. C (2006). Unrecognized trauma and PTSD among public mental health consumers with chronic and severe mental illness. Community Mental Health Journal.

[CIT0015] Davies-Netzley S, Hurlburt M. S, Hough R. L (1996). Childhood abuse as a precursor to homelessness for homeless women with severe mental illness. Violence and Victims.

[CIT0016] Dorrepaal E, Thomaes K, Smit J. H, van Balkom A. J. L. M, Hoogendoorn A. W, Veltman D. J (2012). Stabilizing group treatment for complex posttraumatic stress disorder related to child abuse based on psychoeducation and cognitive behavioural therapy: A multisite randomized controlled trial. Psychotherapy and psychosomatics.

[CIT0017] Dorrepaal E, Thomaes K, Smit J. H, van Balkom A. J. L. M, van Dyck R, Veltman D. J (2010). Stabilizing group treatment for complex posttraumatic stress disorder related to childhood abuse based on psycho-education and cognitive behavioral therapy: A pilot study. Child Abuse and Neglect.

[CIT0018] Draijer N, Boon S (1990). Child sexual abuse and/or physical abuse, dissociation and other trauma-related symptoms within a clinical population.

[CIT0019] Ford J. D, Fournier D (2007). Psychological trauma and post-traumatic stress disorder among women in community mental health aftercare following psychiatric intensive care. Journal of Psychiatric Intensive Care.

[CIT0020] Ford J. D, Stockton P, Kaltman S, Green B. L (2006). Disorders of extreme stress (DESNOS) symptoms are associated with type and severity of interpersonal trauma exposure in a sample of healthy young women. Journal of Interpersonal Violence.

[CIT0021] Friedl M. C, Draijer N (2000). Dissociative disorders in Dutch psychiatric inpatients. American Journal of Psychiatry.

[CIT0022] Gaudiano B. A, Zimmerman M (2010). The relationship between childhood trauma history and the psychotic subtype of major depression. Acta Psychiatrica Scandinavica.

[CIT0023] Goldberg J. F, Garno J. L (2005). Development of posttraumatic stress disorder in adult bipolar patients with histories of severe childhood abuse. Journal of Psychiatric Research.

[CIT0024] Goodman L. A, Thompson K. M, Weinfurt K, Cod S, Acker P, Mueser K. T (1999). Reliability of reports of violent victimization and posttraumatic stress disorder among men and women with serious mental illness. Journal of Traumatic Stress.

[CIT0025] Grant D. M, Beck J. G, Marques L, Palyo S. A, Clapp J. D (2008). The structure of distress following trauma: Posttraumatic disorder, major depressive disorder, and generalized-anxiety disorder. Journal of Abnormal Psychology.

[CIT0026] Griffin M. G, Resick P. A, Waldrop A. E, Mechanic M. B (2003). Participation in trauma research: Is there evidence of harm?. Journal of Traumatic Stress.

[CIT0027] Herman J. L (1992). Complex PTSD: A syndrome in survivors of prolonged and repeated trauma. Journal of Traumatic Stress.

[CIT0028] Herman J. L, Perry J. C, van der Kolk B.A (1989). Childhood trauma in borderline personality disorder. American Journal of Psychiatry.

[CIT0029] Janssen I, Krabbendam L, Bak M, Hanssen M, Vollebergh W, de Graaf R (2004). Childhood abuse as a risk factor for psychotic experiences. Acta Psychiatrica Scandinavica.

[CIT0030] Kauer-Sant’ Anna M, Tramontina J, Andreazza A. C, Cereser K, da Costa S, Santin A (2007). Traumatic life events in bipolar disorder: Impact on BDNF levels and psychopathology. Bipolar Disorders.

[CIT0031] Kessler R. C, Barker P. R, Colpe L. J, Epstein J. F, Gfroerer J. C, Hiripi E (2003). Screening for serious mental illness in the general population. Archives of General Psychiatry.

[CIT0032] Kessler R. C, Berglund P, Demler O, Jin R, Walters E. E (2005a). Lifetime prevalence and age-of-onset distributions of DSM-IV disorders in the National Comorbidity Survey Replication. Archives of General Psychiatry.

[CIT0033] Kessler R. C, Chiu W. T, Demler O, Merikangas K. R, Walters E. E (2005b). Prevalence, severity, and comorbidity of 12-month DSM-IV disorders in the National Comorbidity Survey Replication. Archives of General Psychiatry.

[CIT0034] Kilcommons A. M, Morrison A. P (2005). Relationships between trauma and psychosis: An exploration of cognitive and dissociative factors. Acta Psychiatrica Scandinavica.

[CIT0035] Leverich G. S, McElroy S. L, Suppes T, Keck P. E, Denicoff K. D, Nolen W. A (2002a). Early physical and sexual abuse associated with an adverse course of bipolar illness. Biological Psychiatry.

[CIT0036] Leverich G. S, Perez S, Luckenbaugh D. A, Post R. M (2002b). Early psychological stressors: Relationship to suicidality and course of bipolar illness. Clinical Neuroscience Research.

[CIT0037] Lewis K. L, Grenyer B. F. S (2009). Borderline personality or complex posttraumatic stress disorder? An update on the controversy. Harvard Review Psychiatry.

[CIT0038] Lommen M. J. J, Restifo K (2009). Trauma and posttraumatic stress disorder (PTSD) in patients with schizophrenia or schizoaffective disorder. Community Mental Health Journal.

[CIT0039] Lu W, Fite R, Kim E, Hyer L, Yanos P. T, Mueser K. T (2008). Cognitive-behavioral treatment of PTSD in severe mental illness. American Journal of Psychiatric Rehabilitation.

[CIT0040] Lysaker P. H, LaRocco V. A (2008). The prevalence and correlates of trauma-related symptoms in schizophrenia spectrum disorder. Comprehensive Psychiatry.

[CIT0041] Maguire C, Mc Cusker C. G, Meenagh C, Mulholland C, Shannon C (2008). Effects of trauma on bipolar disorder: The mediational role of interpersonal difficulties and alcohol dependence. Bipolar Disorders.

[CIT0042] Marcello S. C, Hilton-Lerro K, Mueser K. T (2009). Cognitive behavioral therapy for posttraumatic stress disorder in persons with psychotic disorders. Clinical Case Studies.

[CIT0043] McCarthy-Jones C, Davidson L (2012). When soft voices die: Auditory verbal hallucinations and a four letter word (love). Mental Health, Religion & Culture.

[CIT0044] Meade C. S, Mc Donald L. J, Graff F. S, Fitzmaurice G. M, Griffin M. L, Weiss R. D (2009). A prospective study examining the effects of gender and sexual/physical abuse on mood outcomes in patients with co-occurring bipolar I and substance use disorders. Bipolar Disorders.

[CIT0045] Morrison A. P, Frame L, Larkin W (2003). Relationship between trauma and psychosis: Review and integration. British Journal of Clinical Psychology.

[CIT0046] Moskowitz A (2011). Schizophrenia, trauma, dissociation, and scientific revolutions. Journal of Trauma & Dissociation.

[CIT0047] Mueser K. T, Goodman L, Trumbetta S. L, Rosenberg S, Osher F. C, Vidaver R (1998). Trauma and posttraumatic stress disorder in severe mental illness. Journal of Consulting and Clinical Psychology.

[CIT0048] Mueser K. T, Rosenberg S. D, Goodman L. A, Trumbetta S. L (2002). Trauma, PTSD, and the course of severe mental illness: In interactive model. Schizophrenia Research.

[CIT0049] Mueser K. T, Rosenberg S. D, Xie H, Jankowski M. K, Bolton E. E, Lu W (2008). A randomized controlled trial of cognitive–behavioral treatment for posttraumatic stress disorder in severe mental illness. Journal of Consulting and Clinical Psychology.

[CIT0050] Mueser K. T, Salyers M. P, Rosenberg S. D, Goodman L. A, Essock S. M, Osher F. C (2004). Interpersonal trauma and posttraumatic stress disorder in patients with severe mental illness: Demographic, clinical, and health correlates. Schizophrenia Bulletin.

[CIT0051] National Institute of Mental Health (1987). Towards a model for a comprehensive community-based mental health system.

[CIT0052] Neria Y, Bromet E. J, Carlson G. A, Naz B (2005). Assaultive trauma and illness course in psychotic bipolar disorder: Findings from the Suffolk county mental health project. Acta Psychiatrica Scandinavica.

[CIT0053] Neria Y, Olfson M, Gameroff M. J, Wickramaratne P, Pilowsky D, Verdeli H (2008). Trauma exposure and posttraumatic stress disorder among primary care patients with bipolar spectrum disorder. Bipolar Disorders.

[CIT0054] Oquendo M, Brent D. A, Birmaher B, Greenhill L, Kolko D, Stanley B (2005). Posttraumatic stress disorder comorbid with major depression: Factors mediating the association with suicidal behavior. American Journal of Psychiatry.

[CIT0055] Pelcovitz D, van der Kolk B, Roth S, Mandel F, Kaplan S, Resick P (1997). Development of a criteria set and a structured interview for disorders of extreme stress (SIDES). Journal of Traumatic Stress.

[CIT0056] Read J, van Os J, Morrison A. P, Ross C. A (2005). Childhood trauma, psychosis and schizophrenia: A literature review with theoretical and clinical implications. Acta Psychiatrica Scandinavica.

[CIT0057] Resnick S. G, Bond G. R, Mueser K. T (2003). Trauma and posttraumatic stress disorder in people with schizophrenia. Journal of Abnormal Psychology.

[CIT0058] Rosenberg S. D, Lu W, Mueser K. T, Jankowski M. K, Cournos F (2007). Correlates of adverse childhood events among adults with schizophrenia spectrum disorders. Psychiatric Services.

[CIT0059] Ross C. A, Keyes B (2004). Dissociation and schizophrenia. Journal of Trauma and Dissociation.

[CIT0060] Ruggeri M, Leese M, Thornicroft G, Bisoffi G, Tansella M (2000). Definition and prevalence of severe and persistent mental illness. British Journal of Psychiatry.

[CIT0061] Sar V (2011). Review article. Epidemiology of dissociative disorders: An overview. Epidemiology Research International.

[CIT0062] Sar V, Kundakci T, Kiziltan E, Yargic I. L, Tutkun H, Bakim B (2003). The axis-I dissociative disorder comorbidity of borderline personality disorder among psychiatric outpatients. Journal of Trauma & Dissociation.

[CIT0063] Schinnar A. P, Rothbard A. B, Kanter R (1990). An empirical literature review of definition of severe and persistent mental illness. American Journal of Psychiatry.

[CIT0064] Seedat S, Stein M. B, Oosthuizen P. P, Emsley R. A, Stein D. J (2003). Linking posttraumatic stress disorder and psychosis. A look at epidemiology, phenomenology, and treatment. The Journal of Nervous and Mental Disease.

[CIT0065] Stein M. B, Walker J. R, Anderson G, Hazen A. L, Ross C. A, Eldridge G (1996). Childhood physical and sexual abuse in patients with anxiety disorders and in a community sample. American Journal of Psychiatry.

[CIT0066] Tucker W. D (2002). How to include the trauma history in the diagnosis and treatment of psychiatric inpatients. Psychiatric Quarterly.

[CIT0067] van den Berg D, van der Gaag M (2012). Treating trauma in psychosis with EMDR: A pilot study. Journal of Behavior Therapy and Experimental Psychiatry.

[CIT0068] van den Bosch L. M. C, Verheul R, Langeland W, van den Brink W (2003). Trauma, dissociation, and posttraumatic stress disorder in female borderline patients with and without substance abuse problems. Australian and New Zealand Journal of Psychiatry.

[CIT0069] van der Hart O, Nijenhuis E. R. S, Steele K (2005). Dissocation: An insufficiently recognized major feature of complex posttraumatic stress disorder. Journal of Traumatic Stress.

[CIT0070] van der Kolk B. A, Roth S, Pelcovitz D, Sunday S, Spinazzola J (2005). Disorders of extreme stress: The empirical foundation of a complex adaptation to trauma. Journal of Traumatic Stress.

[CIT0071] van Gerven M, van der Hart O, Nijenhuis E. R. S, Kuipers T (2002). Psychosis, trauma, and trauma related psychopathology. Tijdschrift voor Psychiatrie.

[CIT0072] van Minnen A, Harned M. S, Zoellner L, Mills K (2012). Examining potential contraindications for prolonged exposure therapy for PTSD. European Journal of Psychotraumatology.

[CIT0073] Yen S, Shea T. M, Battle C. L, Johnson D. M, Zlotnick C, Dolan-Sewell R (2002). Traumatic exposure and posttraumatic stress disorder in borderline, schizotypal, avoidant, and obsessive-compulsive personality disorders: Findings from the collaborative longitudinal personality disorders study. The Journal of Nervous and Mental Disease.

[CIT0074] Zimmerman M, Mattia J. I (1999). Psychotic subtyping of major depressive disorder and posttraumatic stress disorder. Journal of Clinical Psychiatry.

[CIT0075] Zlotnick C, Johnson D. M, Yen S, Battle C. L, Sanislow C. A, Skodol A. E (2003). Clinical features and impairment in women with borderline personality disorder (BPD) with posttraumatic stress disorder (PTSD), BPD without PTSD, and other personality disorders with PTSD. The Journal of Nervous and Mental Disease.

[CIT0076] Zlotnick C, Mattia J. I, Zimmerman M (2001). Clinical features of survivors of sexual abuse with major depression. Child Abuse and Neglect.

